# Structural Characterization and Immunobiological Activity of Polysaccharides from Astragalus Oyster Mushroom

**DOI:** 10.3390/molecules28135280

**Published:** 2023-07-07

**Authors:** Qiuxia Meng, Yu Niu, Rongrong Wang, Wei Niu, Lizhen Zhang

**Affiliations:** 1 Institute of Eco-environment and Industrial Technology, Shanxi Agricultural University, Taiyuan 030031, China; qiuxia_meng@sxau.edu.cn; 2 College of Agricultural Economics and Management, Shanxi Agricultural University, Taiyuan 030006, China; 3 School of Life Sciences, Shanxi University, Taiyuan 030006, China; 4 Financial and Assets Department, Shanxi Agricultural University, Taigu 030801, China

**Keywords:** Hengshan Astragalus oyster mushroom, polysaccharide, immunomodulation

## Abstract

When added to mushroom growing substrates, edible and medicinal herbs affect the mushrooms’ nutritional and medicinal value. In this study, polysaccharides (P^0^OP-I and P^15^OP-I) were extracted and purified from oyster mushrooms grown on substrates supplemented with 0% and 15% Astragalus roots (P^0^ and P^15^), respectively, and their chemical structure and immunobiological activities were compared. P^15^OP-I and P^0^OP-I were extracted using ultrasound-assisted hot water and deproteinized with the Sevage method, depigmented with 30% H_2_O_2_, desalted with dialysis, and purified using DEAE-52 cellulose and Sephadex G-100 dextran column chromatography. The molecular weight of P^0^OP-I and P^15^OP-I was 21,706.96 and 20,172.65 Da, respectively. Both were composed of monosaccharides *D*-mannose, galacturonic acid, *D*-glucose, *D*-galactose, and *L*-arabinose but in different molar ratios, and both were connected by a pyranoside linkage. P^15^OP-I consisted of higher contents of mannose, glucose, galactose and arabinose and lower content of galacturonic acid as compared to P^0^OP-I. Both P^0^OP-I and P^15^OP-I induced NO and TNF-α production but did not show cytotoxic effect or induce ROS generation in RAW264.7 cells. P^15^OP-I showed a stronger ability to promote NO and TNF-α production relative to P^0^OP-I. In vitro experiments showed that the immunomodulatory activity of P^0^OP-I and P^15^OP-I in RAW264.7 macrophages were mediated by the JNK/MAPK, Erk/MAPK, and NF-κB signaling pathways. The results would be helpful for elucidation of the health promoting mechanism of Astragalus oyster mushrooms as a source of neutraceuticals.

## 1. Introduction

Oyster mushrooms (*Pleurotus ostreatus*) belong to the Basidiomycete division of the Eumycophyta and have been cultivated and consumed as food for thousands of years. The nutritional and medicinal value of oyster mushrooms lies in the various bioactive macromolecules they contain, such as polysaccharides, glycoproteins, flavonoids, polyphenols, and fatty acids [1]. Recent studies have found that polysaccharides play a major role in the biological activity of mushrooms [2] and that mushroom polysaccharides exhibit markable biological activities, including anti-oxidation, hypoglycemic, immunoenhancement, anti-fatigue, and anti-cancer activities [3,4]. Increasing attention has been paid to the structural characteristics and biological activities of mushroom polysaccharides [5].

To date, there have been a few studies implicating the structural elucidation and immunomodulatory activity of the polysaccharides from *P. ostreatus*. Sun and co-workers reported the structure feature of a water-soluble polysaccharide (POP) purified from *P. ostreatus* and its enhancing effect on lymphocyte proliferation induced by concanavalin A (ConA)- or lipopolysaccharide (LPS), and suggested that POP could be a potential immunostimulating agent for use in functional foods or medicine [6]. Two selenium-enriched polysaccharides, Se-POP-21 and Se-POP-3, have been isolated from *P. ostreatus* and explored for their structure and anti-tumor and immunomodulatory activities [7,8]. Meanwhile, some researchers pay more attention to the anti-tumor activities of the polysaccharide from the fungus. An alkali-extracted polysaccharide (WPOP-N1) from the fruiting bodies of *P. ostreatus* was isolated and found to activate macrophages through NF-*κ*B signaling pathway, and thus exhibited anti-tumor effects via this immunostimulating activity [9]. The polysaccharide POMP2 isolated from the mycelia of *P. ostreatus* was found to have a remarkable inhibitory effect on the BGC-823 human gastric cancer cells in vitro and in vivo [10]. A crude polysaccharide extract from the fruiting bodies of *P. ostreatus* was reported to significantly decrease tumor cell metastasis and increase the survival period in mice models of H22 malignant ascites via downregulation of genes Foxp3 and Stat3 and secretion of immunological factors such as IL-2, TNF α, and INF γ [11]. These studies provide valuable reference for the upcoming researchers, and the structure and bioactivities, especially the immunomodulatory effect of *P. ostreatus* polysaccharides, warrant further investigation.

When grown on different substrates, the composition ratio, biological activity, and nutritional value of macromolecular components in mushrooms change significantly [12,13,14]. Astragali Radix, or Astragalus for short, is the dried root of *Astragalus membranaceus* (Fisch.) Bge.var. *mongholicus* (Bge.) Hsiao or *Astragalus membranaceus* (Fisch.) Bunge [15]. It is used as medicine and food in the improvement of health and treatment of various disease [16]. Various scientific literatures show that the supplementation of the mushroom-growing substrates with herbs have simultaneous edible and medicinal value, such as Astragalus and *Codonopsis pilosula* enhancing the nutritional value of edible fungi [14,17,18]. Hengshan Astragalus (also known as “Zheng Bei Qi”, meaning Astragalus of geo-authentic origin), which is produced on Hengshan Mountain, Hunyuan County, Shanxi Province, is a widely used traditional Chinese herb and nutraceutical [19]. In Datong City, Shanxi Province, Hengshan Astragalus is added to the cultivation substrate of oyster mushrooms to produce a new type of edible fungus with high nutritional and health promoting value, named Hengshan Astragalus oyster mushroom (also known as “Bei Qi Gu”). Although the Hengshan Astragalus oyster mushroom has existed for over 20 years and is consumed as a nutraceutical, its bioactive macromolecules have been minimally explored. In recent years, Hengshan Astragalus has been used as the substrate of *Lentinus edodes* (shiitake mushroom) and increased its polysaccharide content. Additionally, mushrooms grown in this way show anti-cancer biological functions [20]. However, the effects of Hengshan Astragalus as an ingredient of growing substrate on the composition and bioactivities of polysaccharides of oyster mushrooms is yet to be investigated.

In this study, polysaccharides from Hengshan Astragalus oyster mushrooms (P^15^) and their counterparts produced on conventional substrate (P^0^) were extracted, and their composition, physical, and chemical properties, and immunobiological activity, were determined. This study provides insights for the further research, development, and application of Hengshan Astragalus oyster mushrooms and their polysaccharides in the fields of food and medicine.

## 2. Results

### 2.1. Extraction and Purification of Polysaccharides

**Figure 1:**
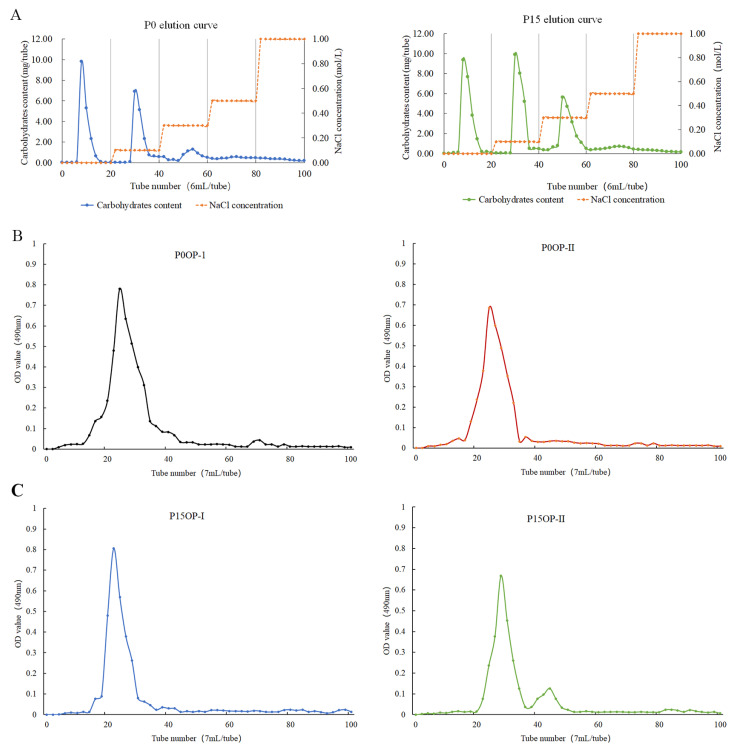
Elution curves of P^0^OP and P^15^OP. (**A**) NaCl gradient elution curves of P^0^OP and P^15^OP in DEAE-52 anion-exchange column chromatography. (**B**) Elution curves of P^0^OP-1 and P^0^OP-2 in Sephadex G-100 gel column chromatography. (**C**) Elution curves of P^15^OP-I and P^15^OP-II in Sephadex G-100 gel column chromatography.

The extraction ratios of crude P^0^ and P^15^ polysaccharides were 23.19% and 25.45%, respectively. After six deproteinization treatments, the protein removal rate of P^0^OP and P^15^OP was 84.52% and 83.97%, respectively, and the polysaccharide retention rate was 70.63% and 68.29%, respectively. The pigment removal rate of P^0^OP and P^15^OP was 68.74% and 67.69%, respectively, and their polysaccharide retention rate was 86.05% and 84.3%, respectively. After dialysis, the P^15^OP solution was slightly yellow, whereas the P^0^OP solution was nearly colorless. Elution curves of the P^0^OP and P^15^OP solutions were obtained using DEAE-52 anion-exchange column chromatography (Figure 1A). P^0^OP yielded two single components, P^0^OP-1 and P^0^OP-2, whereas P^15^OP yielded three single components, P^15^OP-1, P^15^OP-2, and P^15^OP-3. The elution curves of P^0^OP-1, P^0^OP-2, P^15^OP-1, and P^15^OP-2 with higher contents were obtained by Sephadex G-100 gel column chromatography (Figure 1B,C), and single components were obtained, denoted as P^0^OP-I, P^0^OP-II, P^15^OP-I, and P^15^OP-II.

### 2.2. Structural Characterization of Polysaccharides

**Figure 2:**
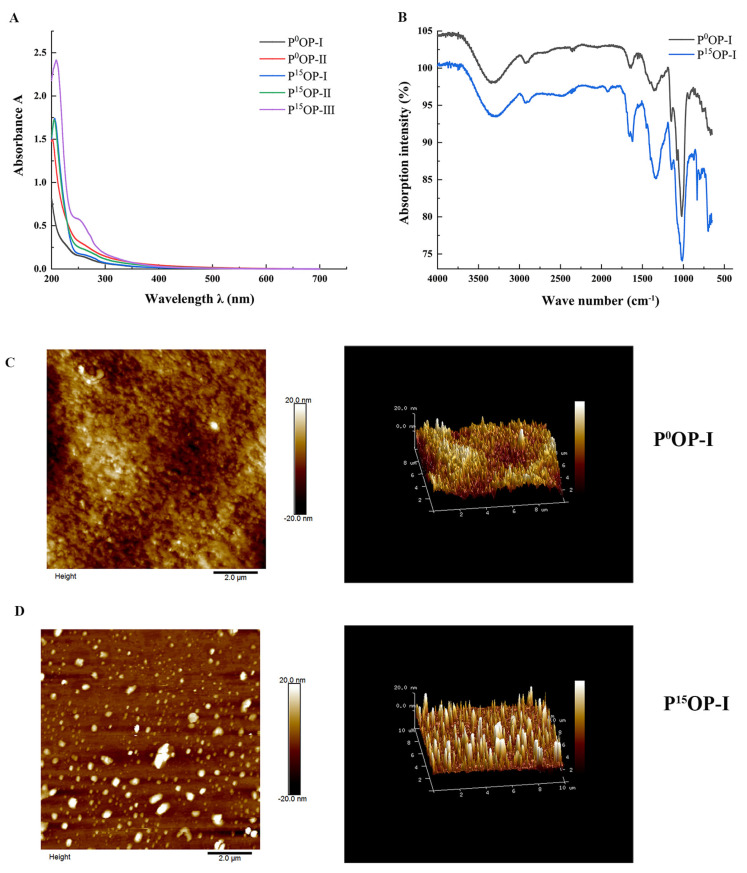
Structural characterization of polysaccharides. (**A**) UV spectra of P^0^OP and P^15^OP. (**B**) Infrared spectra of P^0^OP-I and P^15^OP-I. (**C**) Plane and stereoscopic images of P^0^OP-I with AFM. (**D**) Plane and stereoscopic images of P^15^OP-I with AFM.

The UV spectra of extracted polysaccharides are shown in Figure 2A. No obvious absorption peaks were observed at 260 or 280 nm for the P^0^OP-I, P^0^OP-Ⅱ, P^15^OP-I, and P^15^OP-Ⅱ samples, suggesting that nucleic acids or proteins were scarcely present. In the freeze-thaw experiments, there was no precipitation in the polysaccharide solution, indicating that the polysaccharides of P^0^ and P^15^ obtained after a series of purification operations were relatively pure.

Figure 2B shows the FT-IR scan images of P^0^OP-I and P^15^OP-I. The infrared spectra of the absorption peaks of the two polysaccharides appeared at almost the same wavenumber, but with different intensities. The strong absorption peaks of the two polysaccharides near 3350 cm^−1^ indicated that hydroxyl and amino groups may have been the functional groups. The weak absorption peak near 2930 cm^−1^ suggested the presence of methylene. An absorption peak appeared near 1650 cm^−1^, indicating the presence of an amide group. The variable-angle oscillation peak of the hydroxyl group in the carboxyl group was observed at 1415 cm^−1^, indicating its presence. The wave number region of 1700–900 cm^−1^ was the molecular fingerprint region of polysaccharides, and the wave numbers of the absorption peaks and their corresponding intensities in different polysaccharides were specific, with obvious absorption characteristics that indicated that P^0^OP-I and P^15^OP-I contained ether groups. The characteristic absorption peaks at 873 cm^−1^ and 803 cm^−1^ indicated the presence of mannose. Considering these four absorption peaks together, it can be inferred that P^0^OP-I and P^15^OP-I were connected by pyranoside bonds.

Figure 2C and Figure 2D show the AFM images of P^0^OP-I and P^15^OP-I. In Figure 2C, P^0^OP-I at 1 mg/mL exhibited irregular spherical or coiled structures and a diameter of approximately 4–5 nm, indicating that P^0^OP-I may have undergone molecular aggregation due to the high polysaccharide concentration. Furthermore, P^0^OP-I did not possess a triple-helical conformation because its height was much lower than 15–50 nm. Figure 2D shows that 1 mg/mL P^15^OP-I had a relatively uniform spherical structure with a diameter of 4–5 nm.

### 2.3. Molecular Weight Distribution (MWD)

**Figure 3:**
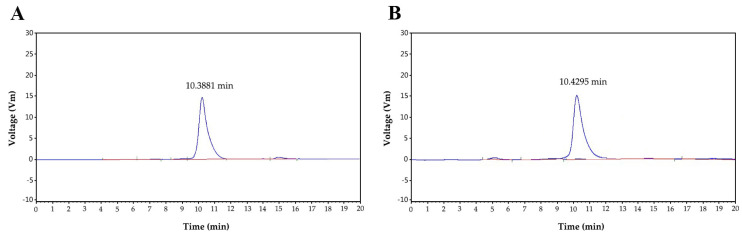
The retention time of P^0^OP-I at 10.3881 min (**A**) and P^15^OP-I at 10.4295 min (**B**), as determined by HPGCP spectrometry.

Figure 3A shows the HPGCP spectrogram of P^0^OP-I. The retention time of P^0^OP-I was 10.3881 min and its relative molecular mass was 21,706.96 Da. Figure 3B shows the HPGCP profile of P^15^OP-I. The retention time of P^15^OP-I was 10.4295 min and its molecular weight was 20,172.65 Da.

### 2.4. Monosaccharide Composition

**Figure 4:**
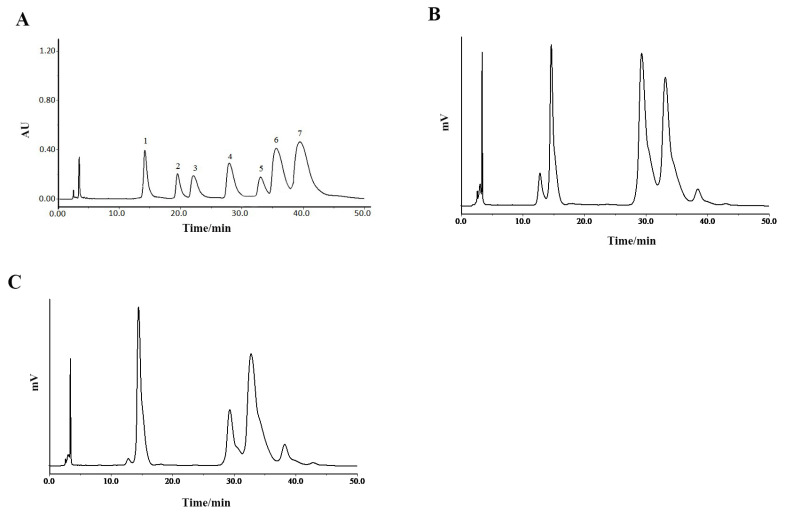
HPLC chromatographic analysis of P^0^OP-I and P^15^OP-I. (**A**) HPLC chromatogram of standard monosaccharide. 1. *D*-mannose; 2. Glucuronic acid; 3. *L*-rhamnose; 4. Galacturonic acid; 5. *D*-glucose; 6. *D*-galactose; 7. *L*-arabinose. (**B**) HPLC chromatogram of P^0^OP-I. (**C**) HPLC chromatogram of P^15^OP-I.

The chromatogram of the mixed monosaccharide standards is shown as Figure 4A. The HPLC spectrogram of P^0^OP-I (Figure 4B) illustrates that its constituent monosaccharides were *D*-mannose, galacturonic acid, *D*-glucose, *D*-galactose, and *L*-arabinose. The molar ratio calculated from the peak area was 22.61:40.94:33.70:2.49:0.26. The HPLC spectrogram of P^15^OP-I (Figure 4C) indicates that its constituent monosaccharides were *D*-mannose, galacturonic acid, *D*-glucose, *D*-galactose, and *L*-arabinose, with a molar ratio of 33.6:14.48:47.23:4.17:0.52. Therefore, the monosaccharide composition of P^0^OP-I and P^15^OP-I was the same but in different molar ratios. Compared with P^0^OP-I, the content of galacturonic acid in P^15^OP-I was lower, and the contents of mannose, glucose, galactose, and arabinose were higher.

### 2.5. In Vitro Immunostimulating Activity

**Figure 5:**
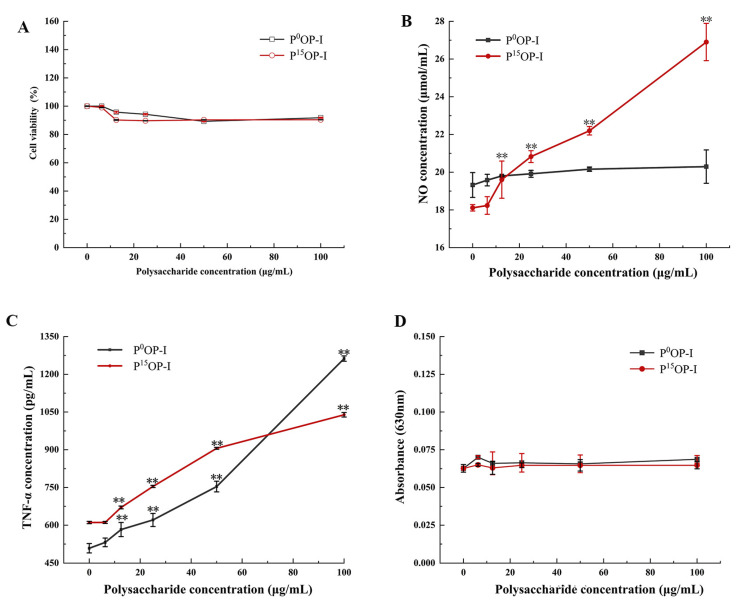
Effect of polysaccharides P^0^OP-I and P^15^OP-I on cell viability and immunocompetence of RAW264.7. (**A**) Cell viability detected by MTT. (**B**) NO release detected by chemical analysis. (**C**) TNF-α production determined using the ELISA method. (**D**) ROS production determined using the nitroblue tetrazolium (NBT) assay. The data are expressed as mean ± SD; ** *p* < 0.01 denote statistically significant difference between the treated group (6.25–100 μg/mL of P^0^OP-I or P^15^OP-I) and the control group (0 µg/mL of P^0^OP-I or P^15^OP-I).

After treatment with 0, 6.25, 12.5, 25, 50, and 100 µg/mL of polysaccharides, the viabilities of RAW264.7 cells were approximately 95% with no significant differences between P^0^OP-I and P^15^OP-I, indicating that these polysaccharides were not cytotoxic to the cells (Figure 5A). At 12.5–100 μg/mL, P^15^OP-I significantly stimulated NO release by RAW264.7 cells in a dose-dependent manner (Figure 5B). At concentrations below 12.5 μg/mL, P^0^OP-I induced greater NO generation than P^15^OP-I, whereas the opposite was true at concentrations above 12.5 μg/mL (Figure 5B). At 12.5–100 μg/mL, P^0^OP-I and P^15^OP-I significantly induced TNF-α secretion in RAW264.7 cells in a dose-dependent manner (Figure 5C). At concentrations below 50 μg/mL, the secretion of TNF-α induced by P^15^OP-I in RAW264.7 cells was significantly higher than that induced by P^0^OP-I, while at a concentration of 100 μg/mL, P^0^OP-I promoted TNF-α secretion more strongly than P^15^OP-I (Figure 5C). At 6.25–100 μg/mL, P^0^OP-I and P^15^OP-I did not affect ROS production by RAW264.7 cells (Figure 5D).

### 2.6. Effect of Cell Signaling Pathway Inhibitors on NO Release and TNF-α Secretion of RAW264.7 Cells Induced by P^0^OP-I and P^15^OP-I

**Figure 6:**
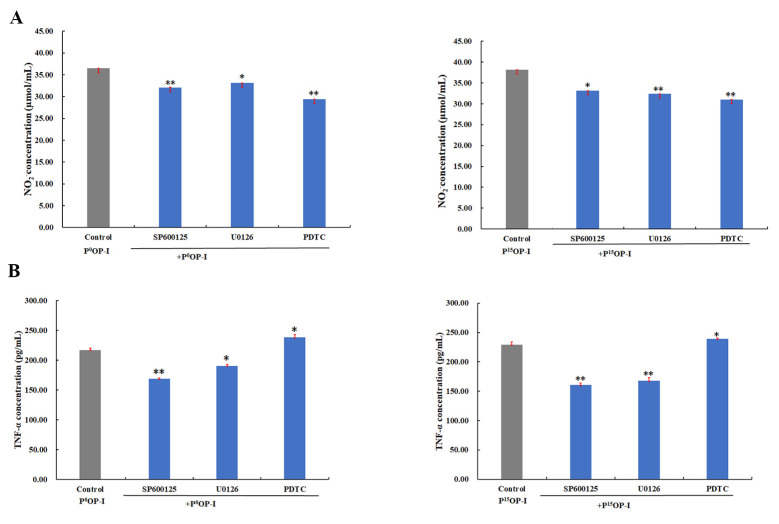
(**A**) The generation of NO induced by P^0^OP-I or P^15^OP-I (gray bars) was lowered by JNK1/2 MAPK inhibitor SP600125, Erk1/2 MAPK inhibitor U0126 and NF-κB activation inhibitor PDTC (blue bars) in RAW264.7 cells. (**B**) The generation of TNF-α induced by P^0^OP-I or P^15^OP-I (gray bars) was lowered by SP600125 and U0126 while enhanced by PDTC (blue bars). The data are expressed as mean ± SD; * *p* < 0.05 and ** *p* < 0.01 denote statistically significant difference between the control group (P^0^OP-I or P^15^OP-I) and the treated group (P^0^OP-I + inhibitor or P^15^OP-I + inhibitor).

As shown in Figure 6A, SP600125 (JNK1/2 MAPK inhibitor), U0126 (Erk1/2 MAPK inhibitor), and PDTC (NF-κB activation inhibitor) blocked the NO release signaling pathway induced by P^0^OP-I and P^15^OP-I, indicating that P^0^OP-I and P^15^OP-I induced NO release and thus activated the JNK/MAPK, Erk/MAPK, and NF-κB signaling pathways in RAW264.7 cells. Figure 6B shows that the secretions of TNF-α induced by P^0^OP-I and P^15^OP-I were significantly inhibited by SP600125 and U0126, which was promoted by PDTC in RAW264.7 cells. These results indicate that the JNK/MAPK, Erk/MAPK, and NF-κB signaling pathways were involved in the secretion of TNF-α promoted by P^0^OP-I and P^15^OP-I in RAW264.7 macrophages.

## 3. Discussion

In this study, the polysaccharides P^15^OP and P^0^OP were purified from Hengshan Astragalus oyster mushrooms and the oyster mushrooms grown on the conventional substrates, respectively. Their spatial structures and monosaccharide components were the same, but the molecular weight and proportion of monosaccharides differed. Moreover, P^15^OP-I generally exhibited stronger inducing activity on the production of NO and TNF-α in RAW264.7 cells than P^0^OP-I, indicating that P^15^OP-I had stronger immunostimulatory activity.

Edible and medicinal herbaceous materials, such as Astragalus and *C. pilosula*, have been used as growing substrate components for edible fungi in China for decades; however, their effects on the chemical constituents and nutritional properties of edible fungi are not well understood. Increasing numbers of studies have shown that plant-derived polysaccharides have immune-enhancing, anti-tumor, and glucose-lowering biological activities [21,22], and Astragalus and oyster mushrooms have been used as extraction materials for bioactive polysaccharides [1,23]. In recent years, various studies have found that adding Astragalus (including raw herbal materials and stems and leaves from agricultural biomass waste) to the growing substrates of oyster mushrooms can improve their contents of active macromolecules and trace elements and bioactivities [14,18,20]. These studies detected beneficial changes in proteins, amino acids, fatty acids, and minerals, but not in polysaccharides, which play a major role in the biological activities of mushrooms. This study revealed, for the first time, that Hengshan Astragalus affects the structure and immunobiological activity of polysaccharides in oyster mushrooms when added to the growing substrate.

It is intriguing that P^15^OP-I, the polysaccharide isolated from Hengshan Astragalus oyster mushrooms, showed stronger immunomodulatory activity by inducing higher levels of NO and TNF-α in macrophages than P^0^OP-I, the polysaccharide isolated from oyster mushrooms grown on conventional substrate. The structural characteristics (such as molecular weight, monosaccharide composition, glycoside bond configuration, spatial structure, and functional groups) of naturally obtained polysaccharides are key factors that determine their biological activities [24,25]. The molecular weight plays an important role in the structure-activity relationship of polysaccharides. A polysaccharide of high molecular weight generally possesses a large excluded volume that enhances the intermolecular interaction between polysaccharide molecules and impedes its uptake by the cells [26]. The molecular weight of P^0^OP-Iand P^15^OP-Iwas 21706.96 Da and 20172.65 Da, respectively. The smaller excluded volume that resulted from a lower molecular weight of P^15^OP-I may be one of the factors responsible for its higher immunomodulating activity in RAW264.7 cells. In addition, the type and proportion of monosaccharides are also closely related to biological activity of polysaccharides. In this study, the polysaccharides isolated from oyster mushrooms were composed of *D*-mannose, galacturonic acid, *D*-glucose, *D*-galactose, and *L*-arabinose, in line with the monosaccharide components described in previous reviews, and the molecular weight was consistent with polysaccharides containing a large proportion of glucose [2]. Our results showed that P^15^OP-I and P^0^OP-I maintained some structural similarities in terms of their monosaccharide composition, spatial structure, and pyranoside linkage, while they differed in terms of the proportion of each monosaccharide. The galacturonic acid content was lower, while those of mannose and glucose, galactose, and arabinose were higher in P^15^OP-I as compared to P^0^OP-I. Lan and colleagues reported that a water-soluble glucose-rich polysaccharide (LPsx) that consisted 95.9% of glucose strongly promoted the production of NO, IL-1β, IL-6 and TNF-α [27]; meanwhile, polysaccharides in longan pulp fermentation with lower molecular weight and higher levels of mannose and arabinose were reported by Hu et al. to exhibit better immunomodulatory activity in activating MAPK and PI3K/Akt signaling pathways [28]. A polysaccharide from *Ganoderma sinense* that was composed of mannose, glucose, and galactose (molar ratio 4.7:27.1:1.0) was also found to increase nitric oxide (NO), TNF-α, and IL-6 production in RAW 264.7 cells [29]. These findings suggest that high levels of glucose, mannose, and arabinose could be crucial for immune-enhancing activity of polysaccharides. Taken together, the addition of Hengshan Astragalus to the growing substrate of oyster mushrooms potentially lowers the molecular weight and increases the ratios of mannose and glucose, galactose, and arabinose of P^15^OP-I, and subsequentially enhanced its ability to induce nitric oxide (NO) and TNF-α generation activity. The specific rules of polysaccharide structure-activity are yet to be set forth and need further exploration nevertheless.

Similar to other mushroom polysaccharides, the anti-tumor activity of oyster mushroom polysaccharides largely depends on their immunomodulatory activity, particularly their ability to activate macrophages [9]. The release of NO and TNF-α was a representative indicator of macrophage activation in the immune activation experiment of mouse macrophage RAW264.7 cells cultured in vitro [30]. Functionally, NO and TNF-α are not only effector molecules of tumor and microbial immunity, but are also regulatory molecules of a variety of immune cells, acting as important immune regulatory molecules in the body [31,32]. This effect of activating macrophage immune activity was also supported by our results, as P^0^OP-I and P^15^OP-I increased NO release and TNF-α production in RAW264.7 cells, indicating that polysaccharides from oyster mushrooms cultivated on different growing substrates have immunobiological activity. Inflammatory activation of macrophages and the production of NO and TNF-α are related to the activation of the transcription factor NF-κB [33]. PDTC (NF-κB activation inhibitor) was used to investigate whether the NF-κB signaling pathway was involved in the pro-activation of RAW264.7 macrophages by P^0^OP and P^15^OP. In this study, NO was significantly reduced when the NF-κB activity was inhibited. Thus, we can conclude that P^0^OP-I and P^15^OP-I promote NO secretion by RAW264.7 macrophages via the NF-κB pathway. However, in RAW264.7 cells co-cultured with P^0^OP-I and P^15^OP-I, the inhibition of NF-κB activation by PDTC resulted in a significant increase in TNF-α production. Further exploration is needed to unveil whether the inactivation of NF-κB caused the compensation of other pathways or if other causes played a part.

MAPKs are protein serine/threonine kinases, including extracellular signal-regulated kinase ½ (ERK1/2), c-Jun N-terminal kinase (JNK), and p38 isoforms. They play a role in translating extracellular stimuli into a wide range of cellular responses and are key pro-inflammatory signaling pathways [34]. Activation of the MAPK pathway is an important signal for macrophage immune activation [35]. In this study, JNK/MAPK and Erk/MAPK inhibitors impaired the promoting effects of P^0^OP-I and P^15^OP-I on the secretion of NO and TNF-α in RAW264.7 cells. These results indicate that JNK/MAPK and Erk/MAPK mediated the immune-enhancing effects of P^0^OP-I and P^15^OP-I in RAW264.7 cells.

In conclusion, both P^0^OP-I and P^15^OP-I are composed of *D*-mannose, galacturonic acid, *D*-glucose, *D*-galactose, and *L*-arabinose in different molar ratios. P^15^OP-I had higher contents of mannose, glucose, galactose, and arabinose, and a lower galacturonic acid content when compared to P^0^OP-I. In vitro experiments showed that P^0^OP-I and P^15^OP-I induced NO and TNF-αgeneration in RAW264.7 cells through the JNK MAPK, Erk/MAPK, and NF-κB signaling pathways.

## 4. Materials and Methods

### 4.1. Oyster Mushroom and Reagents

In this experiment, oyster mushrooms cultivated on a substrate supplemented with 15% Astragali Radix (P^15^) and on conventional substrate with 0% Astragali Radix (P^0^) were the raw materials for polysaccharide extraction. Astragali Radix was purchased from a herb grower on Hengshan Mountain, Hunyuan County, Shanxi Province. The conventional substrate was composed of cotton seed hull (89.3% and 74.3% for P^0^ and P^15^, respectively), corn (7.4%), lime powder (2.2%), phosphatic fertilizer (1.1%), urea (0.2%), bentonite (0.1%), and selenate (0.004%). The Astragalus root was dried, pulverized into 2 mm-diameter particles, and used to replace 15% of the corncob powder in the cultivation substrate. The growth conditions were identical for both P^0^ and P^15^.

Papain, bovine serum albumin (BSA), DEAE-52, and Sephadex G-100 were acquired from SolarBio (Beijing, China). The reference monosaccharides were purchased from Balinway Technology Co. Ltd. (Beijing, China). 1-Phenyl-3-methyl-5-pyrazolone (PMP) and trifluoroacetic acid (TFA) were obtained from Tokyo Chemical Industry Co., Ltd. (Tokyo, Japan). Dimethyl sulfoxide (DMSO) was purchased from Shanghai Yien Chemical Technology Co. Ltd. (Shanghai, China). Inhibitors were purchased from APExBIO Technology LLC (Houston, TX, USA). Apart from acetonitrile, which was of high-performance liquid chromatography (HPLC)-grade, all solvents and chemicals used were analytically pure.

### 4.2. Extraction and Purification of Polysaccharide

#### 4.2.1. Extraction and Purification

Dried and powdered P^15^ or P^0^ was immersed in 75% ethanol for 6 h at a liquid-solid ratio of 1:10 and were then subjected to circumfluence in a water bath for 5 h to remove lipids and phenols. The residue was collected via vacuum suction filtration and was air-dried. The defatted P^15^ or P^0^ powder was then immersed in deionized water (W:V = 1:30) and ultrasonically treated at 100 W for 20 min. Immersion extraction was performed at 85 °C in a water bath for 2 h. The aqueous extract was then centrifuged at 4000 r/min for 15 min and the supernatant was collected. The centrifuged residue underwent the same extraction process, and the supernatants were pooled and rotationally concentrated at 80 °C to obtain crude extraction solutions of P^0^OP and P^15^OP.

The crude extract was deproteinized following the Savage method, depigmented with 30% H_2_O_2_, and desalinated in a 3500U dialysis bag. Crude polysaccharides were obtained by vacuum freeze-drying. The polysaccharide content was measured following the phenol-sulfuric acid method, and the protein content was determined following the Coomassie brilliant blue method. The polysaccharide yield (%), deproteinization rate (%), polysaccharide retention rate (%), and depigmentation rate (%) were calculated using Equations (1)–(4), respectively.

(1) $$\mathrm{Yield}\;\mathrm{of}\;\mathrm{polysaccharide}\;\left(\%\right)=\frac{N\times a\times 100}{B\times 1000}\times 100\%$$

where “N” is the concentration of glucose in the sample (mg/mL), “B” is the weight of P0 or P15 powder, and “a” is the dilution factor.

(2) $$\mathrm{Deproteinization}\;\mathrm{rate}\;\left(\%\right)=\frac{P-P^{\prime }}{P}\times 100\%$$

where “P” and “$\;P^{\prime }$” are the protein contents of the extraction solution before and after deproteinization, respectively.

(3) $$\mathrm{Polysaccharide}\;\mathrm{retention}\;\mathrm{rate}\;\left(\%\right)=\frac{G^{\prime }}{G}\times 100\%$$

where “G” and “$\;G^{\prime }$ are the glucose contents of the extraction solution before and after deproteinization, respectively.

(4) $$\mathrm{Depigmentation}\;\mathrm{rate}\;\left(\%\right)=\frac{A_{S}-A_{S}\prime }{A_{S}}\times 100\%$$

where “As” and “${\mathrm{As}}^{\prime }$” are the absorbance values before and after deproteinization, respectively.

#### 4.2.2. DEAE-52 Cellulose and Sephadex G-100 Gel Chromatography

The deproteinized, depigmented, and desalinized P^0^OP or P^15^OP extraction solutions (15 mg/mL, 10 mL) were filtered through a 0.45 μm ultrafilter and loaded onto the top of a DEAE-52 column (1 cm × 50 cm). The column was gradient-eluted with 0–1.0 mol/L NaCl solution at a flow rate of 0.7 mL/min. The elute was collected at intervals of 10 min/tube, and 20 tubes were collected for each elute concentration. The content of polysaccharide in the tubes was measured using phenol-sulfuric acid method and the absorbance was detected at 490 nm. The elution curve was plotted and the elutes were collected, dialyzed, and freeze-dried based on the peaks that appeared in the elution curve to yield the polysaccharide fractions P^0^ and P^15^. The fractions were then prepared into a 20 mg/mL aqueous solution. An aliquot of 5 mL of the above solution was filtered through a 0.45-μm ultrafilter and then subjected to Sephadex G-100 gel chromatography (1 cm × 50 cm) to isolate the polysaccharides. The column was eluted using distilled water at a flow rate of 0.7 mL/min. The elution curve was plotted based on the absorbance at 490 nm. The elutes were then collected, dialyzed, and freeze-dried based on the peaks that emerged in the elution curve to obtain the purified polysaccharides of P^0^ and P^15^, i.e., P^0^OP-I, P^0^OP-Ⅱ, P^15^OP-I, and P^15^OP-Ⅱ.

### 4.3. Structural Identification of Polysaccharides

#### 4.3.1. Purity Testing

The purified P^0^OP-I, P^0^OP-Ⅱ, P^15^OP-I, and P^15^OP-Ⅱ polysaccharide solutions were subjected to full-wavelength ultraviolet (UV) scanning to observe whether there were peaks indicating impurities in the samples, and then subjected to freezing and thawing tests to determine whether precipitation occurred.

#### 4.3.2. Infrared Spectrum (FT-IR) Scanning

The dried P^0^OP-I and P^15^OP-I samples (1 mg) were prepared as KBr disks and scanned between 4000 and 500 cm^−1^ by infrared spectrometry. The functional groups of the polysaccharides were determined by analyzing their characteristic absorption wavelengths.

#### 4.3.3. Atomic Force Microscope (AFM)

P^0^OP-I or P^15^OP-I was dissolved in deionized water to prepare a 10 μg/L solution. A mica sheet (Φ 1 cm^2^) was split, immediately loaded with the P^0^OP-I or P^15^OP-I solution, and naturally dried in air. The apparent morphology of the polysaccharide molecules was investigated using an AFM in air. A commercial silicon nitride cantilever (length: 115 µm, width: 25 µm) with a spring constant of 0.4 N/m was used.

#### 4.3.4. Distribution of Average Molecular Weight

The homogeneity and molecular weight of the polysaccharides were tested using high-performance gel permeation chromatography (HPGPC). P^0^OP-I or P^15^OP-I was dissolved in deionized water to prepare a 5 mg/mL solution. The column temperature was 35 °C. Distilled water was used as the column eluent at a flow rate of 0.5 mL/min. Dextran reference substances of various molecular weights (T-180, T-2 500, T-4 600, T-7 100, and T-10 000) were used to construct the calibration curve. The standard curve was plotted using the following equation:

$$\mathrm{lgMw}=-0.7959t+12.606,\;R^{2}\;=0.999.$$

#### 4.3.5. HPLC Analysis of Monosaccharide Composition

The dried polysaccharide (10 mg) was hydrolyzed with 2.0 mol/L trifluoroacetic acid in a glass tube at 110 °C for 3 h; the trifluoroacetic acid was removed by evaporation using methanol under reduced pressure. The hydrolysate was prepared as a 1 mL deionized aqueous solution. Each reference monosaccharide was dissolved in ultrapure water to obtain mixed standard solutions. Approximately 0.2 mL of the hydrolysate was mixed with 1-phenyl-3-methyl-5-pyrazolone (PMP) (prepared from 0.2 mL of 0.3 M aqueous sodium hydroxide and 0.24 mL of 0.5 M methanol) and placed in a water bath for 70 min (70 °C, 300 r/min). The mixture was then cooled to 20 to 25 °C and neutralized by adding 200 *μ*L of 0.3 M hydrochloric acid solution. The solution was extracted with chloroform (1 mL), centrifuged thrice at 5000 rpm for 5 min, and then filled to 1 mL with ultrapure water. An aqueous layer (at least 0.4 mL) was collected and passed through a 0.45 μm filter for HPLC measurements. The HPLC analysis was conducted using an E2695 HPLC system (Bangxin Electronic Technology. Co., Ltd., Suzhou, China) equipped with ODS-2 C_18_ column (250 mm × 4.6 mm, 5 μm particle size, ThermoFisher Scientific, Waltham, MA, USA). The separations were performed at 35 °C with elution at a flow rate of 1mL/min with a mobile phase consisted of 0.1 mol/L phosphate buffer solution (pH 6.0) and acetonitrile (85:15, *V*/*V*). The volume of each specimen was 10 μL. The absorbance was detected at λ = 245 nm.

### 4.4. Cell Culture

Mouse macrophage (RAW264.7) cells were provided by the Institute of Biomedicine, Shanxi University. The cells were maintained in Dulbecco’s modified eagle medium (DMEM) in an incubator with 5.0% CO_2_ at 37 °C and were passaged when the cell confluence reached approximately 90%. They were used for the following experiments after being passaged three times. To explore the roles of MAPKs and NF-κB, RAW264.7 cells were pre-treated with 50 µL of inhibitor solution (U0126 for Erk1/2 MAPK, SP600125 for JNK1/2 MAPK, and PDTC for NF-κB) at 20 µmol/L for 1 h and then treated for another 24 h with the P^0^OP-I or P^15^OP-I solution. The cells and culture supernatants were then collected for subsequent experiments.

### 4.5. Cell Viability

RAW264.7 cells were seeded in a 96-well plate and cultured for 24 h. P^0^OP-I or P^15^OP-I solution prepared in DMEM was added to reach the final concentrations (100, 50, 25, 12.5, 6.25, and 0 µg/mL) and maintained in an incubator at 37 °C with 5.0% CO_2_ for 24 h. Cell viability was measured using the MTT assay with an MTT kit (Solarbio, Beijing, China). A volume of 10 µL of 0.5% MTT solution was added to each well and then incubated for 4 h. A volume of 100 µL of DMSO was used to dissolve the formazan. The absorbance of each well was measured at 570 nm using a Cytation-5 cell imaging multifunctional detection system (BioTek, Winooski, VT, USA).

### 4.6. NO and TNF-α Generation

RAW264.7 cells were seeded in a 96-well cell-culture plate and treated with 100 µL of the P^0^OP-I or P^15^OP-I solution at gradient concentrations (100, 50, 25, 12.5, 6.25, and 0 µg/mL) for 24 h. NO in the culture supernatant was detected using an NO Reagent Assay Kit (Elabscience Biotechnology Co., Ltd., Wuhan, China). TNF-α in the culture supernatant was detected following the ELISA method with a Mouse ELISA kit (Solarbio, Beijing, China). Detection was performed following the manufacturers’ protocols.

### 4.7. ROS Production

RAW264.7 cells were seeded in 24-well plates and cultured for 24 h. The cells were treated with 450 µL of the P^0^OP-I or P^15^OP-I at 100, 50, 25, 12.5, 6.25, and 0 µg/mL that dissolved in Hank’s balanced salt solution (HBSS). After 10 min of treatment, 50 µL of 2.5% nitroblue tetrazolium (NBT) solution was added to each well and the culture was continued for 1 h. The unreacted NBT solution was removed by gentle washing with HBSS. To each well, 600 µL of DMSO was added to dissolve formazan, along with 700 µL of KOH solution (2 mol/L). The absorbance of each well was measured at 630 nm (OD630) using a Cytation-5 cell imaging multifunctional detection system (BioTek, USA).

### 4.8. Statistical Analysis

Three independent parallel experiments were conducted. One-way ANOVA or Student’s *t*-test was used to test for significance using Office Excel 2007 (Microsoft, Redmond, WA, USA) and SPSS 20.0 (IBM Co., Armonk, NY, USA).

## Data Availability

Not applicable.

## Sample Availability

Samples of the compounds P^0^OP-I or P^15^OP-I are available from the authors.
